# Life stage-specific inbreeding depression in long-lived *Pinaceae* species depends on population connectivity

**DOI:** 10.1038/s41598-021-88128-4

**Published:** 2021-04-23

**Authors:** Jon Ahlinder, Barbara E. Giles, M. Rosario García-Gil

**Affiliations:** 1 Division of CBRN Defence and Security, Swedish Defence Research Agency, 901 82 Umeå, Sweden; 2 Department of Ecology and Environmental Science, Umeå University, 901 87 Umeå, Sweden; 3 Department of Forest Genetics and Plant Physiology, Swedish University of Agricultural Sciences, 901 87 Umeå, Sweden

**Keywords:** Evolution, Genetics, Plant sciences

## Abstract

Inbreeding depression (ID) is a fundamental selective pressure that shapes mating systems and population genetic structures in plants. Although it has been shown that ID varies over the life stages of shorter-lived plants, less is known about how the fitness effects of inbreeding vary across life stages in long-lived species. We conducted a literature survey in the *Pinaceae*, a tree family known to harbour some of the highest mutational loads ever reported. Using a meta-regression model, we investigated distributions of inbreeding depression over life stages, adjusting for effects of inbreeding levels and the genetic differentiation of populations within species. The final dataset contained 147 estimates of ID across life stages from 41 studies. 44 Fst estimates were collected from 40 peer-reviewed studies for the 18 species to aid genetic differentiation modelling. Partitioning species into fragmented and well-connected groups using Fst resulted in the best way (i.e. trade-off between high goodness-of-fit of the model to the data and reduced model complexity) to incorporate genetic connectivity in the meta-regression analysis. Inclusion of a life stage term and its interaction with the inbreeding coefficient (F) dramatically increased model precision. We observed that the correlation between ID and F was significant at the earliest life stage. Although partitioning of species populations into fragmented and well-connected groups explained little of the between-study heterogeneity, the inclusion of an interaction between life stage and population differentiation revealed that populations with fragmented distributions suffered lower inbreeding depression at early embryonic stages than species with well-connected populations. There was no evidence for increased ID in late life stages in well-connected populations, although ID tended to increase across life stages in the fragmented group. These findings suggest that life stage data should be included in inbreeding depression studies and that inbreeding needs to be managed over life stages in commercial populations of long-lived plants.

## Introduction

Ever since Darwin investigated the effects of self-fertilising and outcrossing mating systems on offspring vigour^1^, evolutionary geneticists have recognized that inbreeding depression (ID), or the reduced viability and/or fecundity of the offspring of related parents, is a primary selective force driving mating system evolution. ID maintains outbreeding and prevents sexual systems from evolving towards exclusive self-fertilization^2,3^. Inbreeding is also a concern for breeders and conservationists because of its negative effects on population performance and survival^4,5^. Whereas the vast majority of studies have focused on shorter-lived plants, considerably less effort has been devoted to studying the effects of ID in long-lived outcrossing species such as trees, and of the work that has been done, almost all have focused on ID in early embryonic stages^4,6,7^. Even fewer studies have addressed the question of how population size and genetic differentiation affect the strengths of ID across life stages of long-lived species. This lack of knowledge is surprising given the long-standing recognition that tree species should be more favourable than short-lived species as models for studying the cumulative life stage effects of inbreeding on ID^4,8^.

Theoretical models have identified mating system (i.e., outcrossing versus selfing) as a key determinant of the timing of population ID^2,9^. Selfers are more often observed to express ID in later life stages (i.e., survival and growth/reproduction) whereas outcrossers exhibit higher ID at either early or both early and late life stages^2,5^. Husband and Schemske^5^ concluded that the early stage ID seen in outcrossing species likely arises from the expression of recessive lethal or strongly deleterious alleles in homozygous form. Early life stage ID should thus be rarer in selfers because homozygotes for deleterious alleles coding for traits expressed at this stage would have been exposed to selection and immediately purged from populations. In contrast, deleterious alleles for genes expressed in later life stages could be maintained in populations as long as they do not affect plant establishment and/or reproduction. Late ID has also been interpreted to be the result of the cumulative effects of smaller fitness reductions^10^ caused by mildly deleterious recessive alleles that are more difficult to purge^5^. The result, given either of these alternative explanations, should be an increase in late life stage ID in both selfing and outcrossing mating systems.

The strength and timing of ID can also be affected through alterations of the mating system induced by past demographic changes. Geographically restricted and isolated populations descended from species with once large, well-connected outcrossing populations would experience decreases in effective population sizes and hence increased consanguineous mating^11^. Relatively soon after population size reduction, progeny would be expected to suffer from severe ID resulting from increased homozygosity for the deleterious recessive alleles that had accumulated in the previously large outbreeding populations^5^. Should inbreeding continue in these small populations, selective elimination of the deleterious alleles (purging) is expected, leading to reduced levels of ID as observed in species with self-fertilising mating systems^2^. Purging may not, however, act effectively on fitness traits that are controlled by many genes of small effect or when *N*_*e*_ becomes very small^3,12^. However, in small populations affected by a longer histories of inbreeding, crossing experiments designed to measure ID in these populations may also fail to detect further reductions in population fitness (ID baseline hypothesis)^13^. This could lead to the erroneous inference that the apparently low levels of ID were caused by purging (purging hypothesis). Although both hypotheses are possible, their effects on the evolution of population mating system will differ. Under the purging hypothesis, the major force counteracting a transmission advantage of selfing is lost and breeding systems will evolve to allow greater levels of inbreeding or selfing, which would not be the case under the ID baseline hypothesis.

Our study focuses on the family *Pinaceae*, which, although self-compatible, tend to be highly outcrossing, effectively resulting in large randomly mating populations^6^. In conifers, most published studies of ID have focused on a single early life stage, most commonly, the high abortion rate of seeds during embryo development^14,15^. Selective embryo abortion in conifers reduces the impact of genetic load caused by nearly lethal recessive mutations that accumulate during the large number of cell divisions that occur before flowering^16^. Two factors contribute to the maintenance of the high frequency of recessive mutations in conifers, namely, effective outcrossing facilitated by long distance pollen flow, greater survival of outcrossed progenies and large effective population sizes. In a review of the literature of ID in conifers, Williams and Savolainen^17^ concluded, in accordance with theoretical predictions, that inbreeding effects were most pronounced at early development stages (see also Koelewijn et al.^18^) although other authors have found increased ID at late developmental stages^19^. Further effort to investigate the distribution of ID across life stages is thus warranted.

Here we investigate the distribution of ID across four life stages in 18 species of the *Pinaceae* collected from 41 individual studies. Unlike Husband and Schemske’s meta-analysis of the timing of ID in plants^5^, our study is performed on species from a single family (*Pinaceae*) that share many biological characteristics such as a predominantly outcrossing mating system, wind pollination, long-distance gene flow and long life-span, thus offering a more homogeneous group that can be subdivided into classes based on their population sizes and levels of genetic differentiation. *Pinaceae* is also characterized by a high lifetime fecundity and large pollen and seed production that permits the abortion of large numbers of inbred seeds without compromising population fitness. A dramatic reduction in population effective size could, however, result in a transition towards a selfing mating system that would permit, after many generations, the purging of genetic loads so characteristic of *Pinaceae* species^20–23^. We aim to answer the following questions in this study: (1) how is ID distributed among different life stages of long-lived tree species? How does the inbreeding coefficient affect the distribution of ID across these life stages? (2) Do species with small isolated populations differ in the magnitude and/or timing of ID from those occupying large, continuous areas of distribution?

## Results

### The final dataset

**Figure 1:**
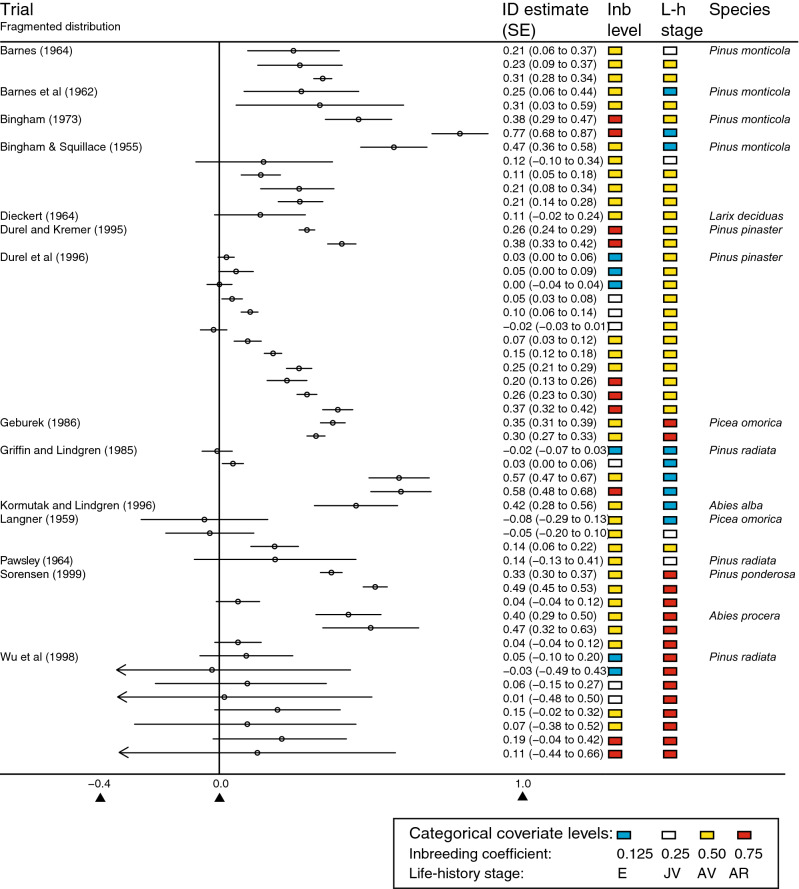
Forest plot of the final dataset including study-specific covariate levels for species with fragmented population distributions. The x-axis shows the level of ID. Life stage and Inbreeding are abbreviated as L-h and Inb.

**Figure 2:**
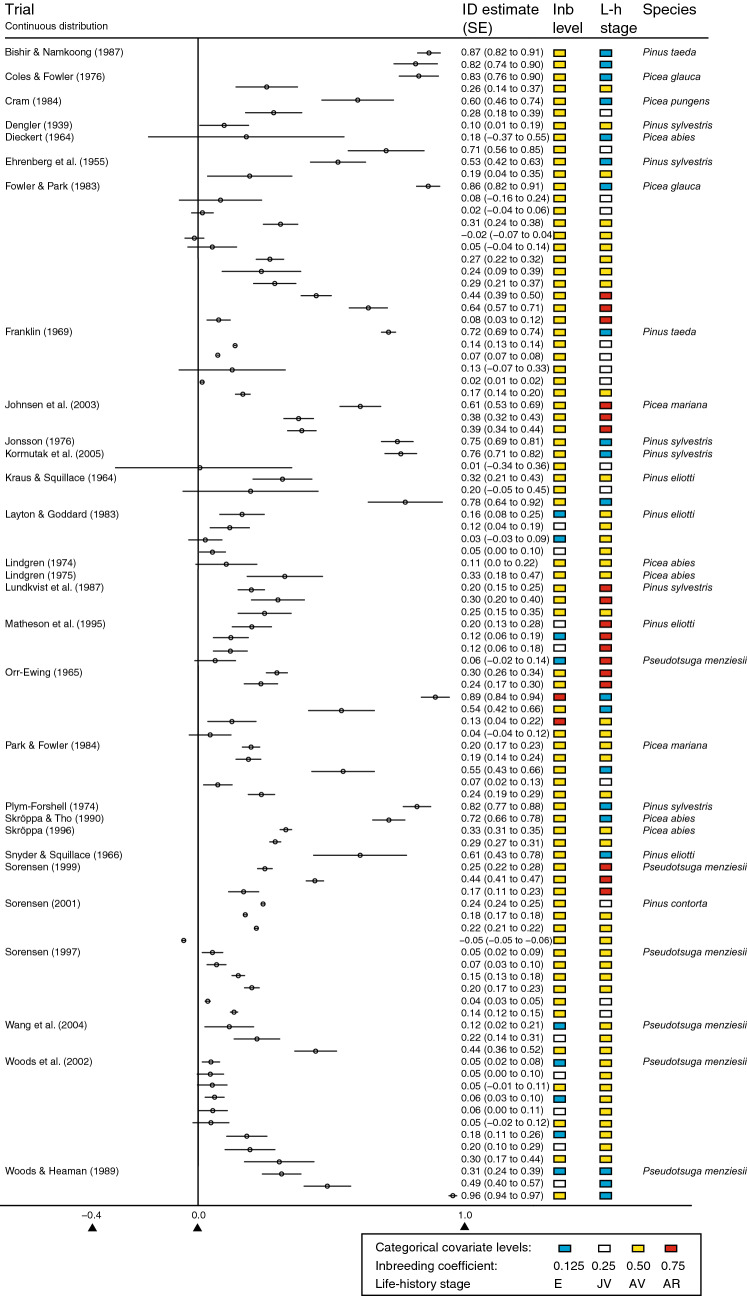
Forest plot of the final dataset including study specific covariate levels for species with well-connected populations. The x-axis shows the level of ID. Life stage and Inbreeding are abbreviated as Life and Inb.

First, ID data for 18 species from five genera (*Pinus*, *Abies*, *Picea*, *Pseudotsuga* and *Larix*) in the *Pinaceae* were obtained from 41 studies (Figs. 1, 2, Fig. S1, Table S1), by using the Web of Science database as well as older reports held in plant breeding institutions. In total, 147 estimates of ID were included in the analysis, which makes this one of the more extensive compilations for long-lived perennial species reported in the literature. The 10th, 50th and 90th percentiles of the collected ID estimates corresponded to IDs of 0.03, 0.20 and 0.707, respectively. In addition to ID estimates and their standard errors (SE), data that could explain variation in ID such as inbreeding coefficients (F) and the life stages of the inbred populations, were collected. The study-specific inbreeding coefficient(s) were obtained from the respective crossing designs performed in each study, resulting in F = {0.125, 0.25, 0.5, 0.75}. For some study-specific configurations (i.e. combinations of life stage, inbreeding coefficient and species fragmentation status), a large number of ID estimates were available: for an inbreeding coefficient of F = 0.5, 99 ID estimates were collected, mostly from self-crossing designs (Fig. S1a); and for the adult vegetative life stage, 69 ID estimates were available (Fig. S1b), in which some ID estimates partly overlapped with the F = 0.5 group. The number of ID estimates within each level of inbreeding coefficient and species distributions were, in some cases, highly unbalanced (Fig. S1a); for example, only two estimates were available for continuously distributed species and F = 0.75. To account for this unbalanced design, a random effects regression model was used to analyse the data.

Prior to carrying out the meta-analysis, we tested the data for homogeneity to investigate whether the ID estimates from the experiments were sufficiently similar to warrant their combination into an overall effect size term (i.e. a single study-level effect) or whether additional study-level data would improve the analysis. Our hypothesis was that the addition of information, such as crossing-designs, life stages and genetic connectivity for each species, would explain a greater portion of variation in ID estimates than consideration of individual study-effects alone. There was strong evidence for heterogeneity in the data (Q = 9014, *p* < 0.001), which supports the addition of the aforementioned species-specific details into the regression analysis. The next step was to decide a suitable way of incorporating the species-specific population sizes and genetic connectivity into the regression analysis.

### Genetic connectivity data is best utilized by dividing the species into two groups according to Fst distribution

To acknowledge the impact of genetic connectivity on ID, three alternative methods were evaluated. To facilitate this analysis, 44 Fst estimates were collected from 40 peer-reviewed studies for all 18 species included in the study (Table 1, Supplementary information). We hypothesised that the genetic connectivity of a species could be manifested through the Fst estimates. In addition, the genetic marker type (SNPs, SSRs and Allozymes/Isozymes) used to estimate Fst were noted for each species. To make Fst estimates easier to compare in a single analysis, we included codominant markers based on nuclear DNA and rejected Fst estimates derived from markers that were either dominant (such as RAPD and AFLP) or based on organellar DNA (chloroplast or mitochondria). In model 1, species were partitioned into two groups according to the area of species distribution and level of patchiness. In model 2, a beta regression model and a k-means clustering algorithm were used to partition the species based on their averaged estimates of Fst. This regression-based approach allowed for inferring the species effects on Fst while adjusting for the different marker types used to estimate Fst in the respective studies. By adjusting for marker type, we believe that the inferred species effects on Fst would be comparable. In model 3, the posterior mean of the species effect on Fst, obtained from the beta regression model, was used as a covariate.

**Table 1:** Species connectivity using the United States Department of Agriculture Natural Resources Conservation Service (http://plants.usda.gov/) for the American species and Agro Forestry Tree Database (http://www.worldagroforestry.org/) for European species.

Species	Latin name	Manual group 1 = cont 2 = fract	Species distribution	k-means group 1 = cont 2 = fract	Reference	*Fst*	Marker type	No of markers	No of pop	Area
Norway spruce	*Picea abies*	1	Eurasia	1	Unger et al. (2012)	0.002	EST-SSR^a^	6	3	Austria
					Achere et al. (2005)	0.009	SSR	25	3	Europe
					Achere et al. (2005)	0.029	AFLP^b^	265	3	Europe
					Chen et al. (2012)	0.05	SNP^c^	445	18	Europe
Western white pine	*Pinus monticola*	2	West of North America, One isolated population in the south	2	Kim et al. (2011)	0.201	AFLP	66	15	Western NA
					Liu et al. (2011)	0.163	SNP	53	7	Western NA
White spruce	*Picea glauca*	1	West/North of North America	1	Namroud et al. (2008)	0.006	SNP	534	6	Québec
Maritime pine	*Pinus pinaster*	2	Western Mediterranean basin, south Europe and Africa and Atlantic cost of Spain, Portugal and France	2	Wahid et al. (2010)	0.120	ncSSR^d^	7	10	Marocco
					Soto et al. (2010)	0.221	cpSSR^e^	6	38	Iberia
					Eveno et al. (2008)	0.150	ncSSR	8	10	Mediterranean coast
						0.137	SNP	302	10	
					Jaramillo-Correa et al. (2015)	0,251	SNP	18^ g^	36	Mediterranean
European Larch	*Larix decidua*	2	Central Europe, in the mountains	1	Wagner et al. (2012)	0.082	SSR	13	18	Central Europe
					Mosca et al. (2012)	0.043	SNP	267	24	Northern Italy
Scots pine	*Pinus sylvestris*	1	Eurasia	1	Soto et al. (2010)	0.070	cpSSR	6	30	Iberia
					Scalfi et al. (2009)	0.080	ncSSR	3	3	Italy
					Kahru et al. (1996)	0.020				
					Dvornyk et al. (2002)	0.017^ h^	SNP	12	3	Finland and Russia
						0.11	SNP	12	4	Finland, Russia and Spain
					Pyhäjärvi et al. (2007)	0.065	SNP	153	4	Europe
Loblolly pine	*Pinus taeda*	1	South east USA Most of it planted after the Great depression	1	Eckert et al. (2010)	0.043	SNP	1730	54	South east UsA
Slash pine	*Pinus elliottii*	1	South east USA. The smallest distribution of the four south USA pines	1	Berg and Hamrick (1997)	0.028	Allozymes	2	16	South east USA
Radiata pine	*Pinus radiata*	2	Cost of California	2	Kahru et al. (2006)	0.140	SSR	19	5	California
					Moran et al. (1988)	0.162	Allozymes	31	5	California
Black pine	*Pinus nigra*	2	North Africa, South Europe and Asia Minor	2	Soto et al. (2010)	0.136	cpSSR	6	14	Iberia
P. pinceana	*Pinus pinceana*	2	Small population size and endemic species of Mexico	2	Leidig et al. (2001)	0.152	Allozymes	27	8	Mexico
Jack pine	*Pinus banksiana*	2	Canada and north-eastern and northern-central USA	1	Saenz-Romero et al. (2001)	0.022	Allozymes	82	14	Wisconsin
					Ye et al. (2002)	0.155	RAPD	39	9	Alberta
Douglas fir	*Pseudotsuga menziesii*	1	Pacific coast of North America	1	Viard et al. (2001)	0.019	cpSSR	11	11	British Columbia
						0.072	RAPD^f^	48		
						0.018	Allozymes	20		
Nobel fir	*Abies procera*	2	West coast of USA	2	Yeh and Hu (2005)	0.112	Allozymes	14	21	Oregon to washington
Ponderosa pine	*Pinus ponderosa*	2	South-west of Canada and Central-west of USA	2	Latta & Mitton (1999)	0.062	Allozymes	15	8	western North America
						1.000	RAPD-mtDNA	4		
						0.652	RAPD-cpDNA	3		
Lodgepole pine	*Pinus contorta*	1	From Rocky Mountain and Pacific coast regions, extending north to the Yukon Territory and south to Baja California, and west to east from the Pacific Ocean to the Black Hills of South Dakota	1	Parchman et al. (2012)	0.008	SNP	97,616	3	Wyoming
Virginia pine	*Pinus virginiana*	1	West Canada, east USA	1	Parker et al. (1997)	0.053	Allozymes	26	19	Eastern USA
Blue spruce	*Picea pungens*	2	Eastern Canada and USA, Western USA	1	Leidig et al. (2006)	0.086	Allozymes	17	4	Western USA
Serbian spruce	*Picea omorica*	2	Serbia and Bosnia and Herzegovina	2	Ballian et al. (2006)	0.261	Isozymes	16	13	Balkan
Black spruce	*Picea mariana*	1	Canada, Northeast USA	1	Wang and Macdonald (1992)	0.010	Allozymes	28	6	Canada
Silver fir	*Abies alba*	1	European mountains		Matusova (1995)	0.015	Allozymes	15	5	Bulgaria
					Mosca et al. (2012)	0.037	SNP	249	37	Italy, Macedonia

References provided in Supplementary information.

^a^Expressed sequence tags—simple-sequence repeats.

^b^Amplified fragment length polymorphism.

^c^Single nucleotide polymorphism.

^d^Nuclear simple-sequence repeats.

^e^Chloroplast simple-sequence repeats.

^f^Random amplification of polymorphic DNA.

^g^Only SNPs associated with climate gradient.

^h^If a single population from Spain was excluded.

In model 1, ten species were placed in the partition corresponding to a more continuous (well-connected) area of distribution, while eight were placed in the patchy partition: mean Fst was 0.192 (standard deviation 0.028) and 0.035 (0.001) for the fragmented and continuous distributions, respectively (Table S1). Although this partitioning was not strictly based on Fst estimates, the resulting partition corresponded well to average Fst estimates.

**Figure 3:**
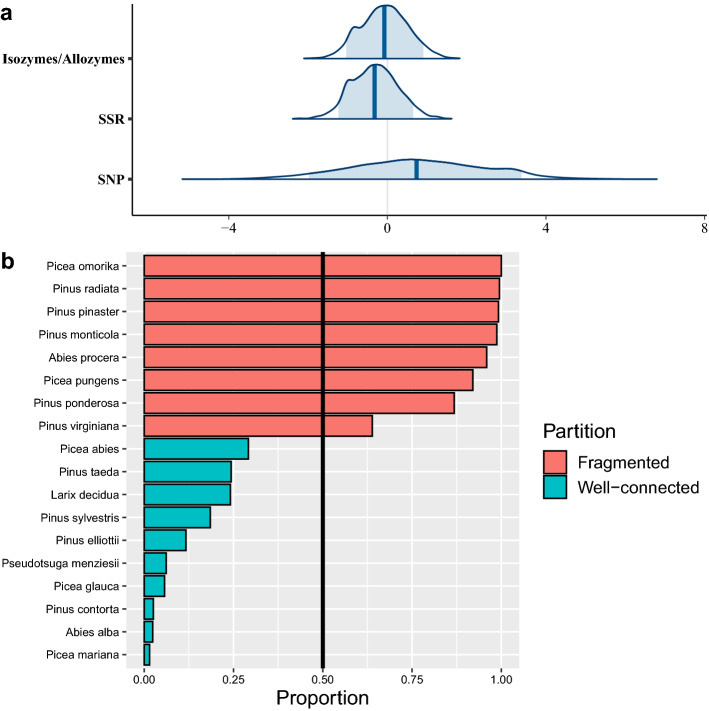
The results of the beta-regression and clustering of the *Pinaceae* species into fragmented or well-connected groups, with: (**a**) inferred posterior distributions of marker types with 90% CI highlighted in blue, and (**b**) model 2 approach to partition the species into two groups, where the Serbian spruce was fixed in the patchy partition (because of the highest average *F*_st_) to avoid label switching problems.

A beta-regression analysis was then carried out to model the effect of marker types and species on observed Fst values using a Bayesian approach. The result of this regression analysis showed less variation in Fst estimates for Isozymes/Allozymes and SSR markers than for SNP-based Fst estimates, which had greater dispersion (Fig. 3a). As the marker type effects were possible to estimate and had a closed form, we were able to compensate for variation in reported Fst values due to marker system. In other words, by correcting for marker type, we could increase the statistical power in the analysis and more precisely estimate species effects on Fst. The k-means clustering of effect size scores of the species predictors in the beta-regression analysis (Fig. 3b) resulted in a partition where, compared to the partitioning in model 1, two species were moved from the fragmented to the continuous group. *Larix decidua* (average Fst of 0.0625) and *Abies alba* (average Fst of 0.026), resulting in partition averages of Fst of 0.241 (0.028) and 0.037 (0.001) for the fragmented and continuous groups, respectively (Fig. 3b). Most uncertainty in the partitioning occurred for Virginia pine (into the patchy group) and Norway spruce (into the continuous group). The average Fst value increased slightly in both partitions when Fst data were utilized for clustering the species, while the within-group variation decreased slightly (results not shown).

Model performances on the ID data were then compared to decide which result best incorporated species connectivity into the analysis. Based on the predictive performance using two model criteria, LOOIC and LOO, model 2 outperformed the other models, although model 1 performed almost equally well (Table 2), which is not surprising given the similarities in species partitioning. This indicates that model 2 represented the best way to incorporate population size and connectivity descriptions of species in the *Pinaceae* family. Model 3, which used average species effects on Fst as a covariate, performed least well. Thus, the meta-regression model was finalized for further analysis of the ID data by using the partitions of species obtained from model 2.

**Table 2:** Model criteria statistics including posterior mean estimates obtained from the RStan analysis.

Model	ELPD LOO	p LOO	LOOIC	*σ*_*y*_
1	54.2	54.0	− 108.4	1.28 (0.16)
2	54.9	52.0	− 109.8	1.27 (0.15)
3	52.1	70.2	− 104.2	1.24 (0.17)

LOO is abbreviation for leave-one-out cross-validation, LOOIC is the LOO information criterion, ELPD is the expected log pointwise predictive density for a new, simulated, dataset based on the inferred posteriors obtained from the RStan analysis, p is a simulation-estimated effective number of parameters. Standard errors are in parentheses. Higher values of elpd indicate a better predictive performance. The posterior standard deviations are shown in parentheses and *σ*_*y*_ is the model residual standard deviation.

To make all ID estimates comparable, we included species and study-specific predictors in all models so that any individual species or study effects on ID were accounted for in the regression analysis. Neither the species nor the study predictors showed clear effects on ID (Figs. S3 and S4, respectively) and all credible intervals (CI) contained zero at any relevant confidence level.

### ID is most pronounced at highest levels of inbreeding and at early life stages

**Figure 4:**
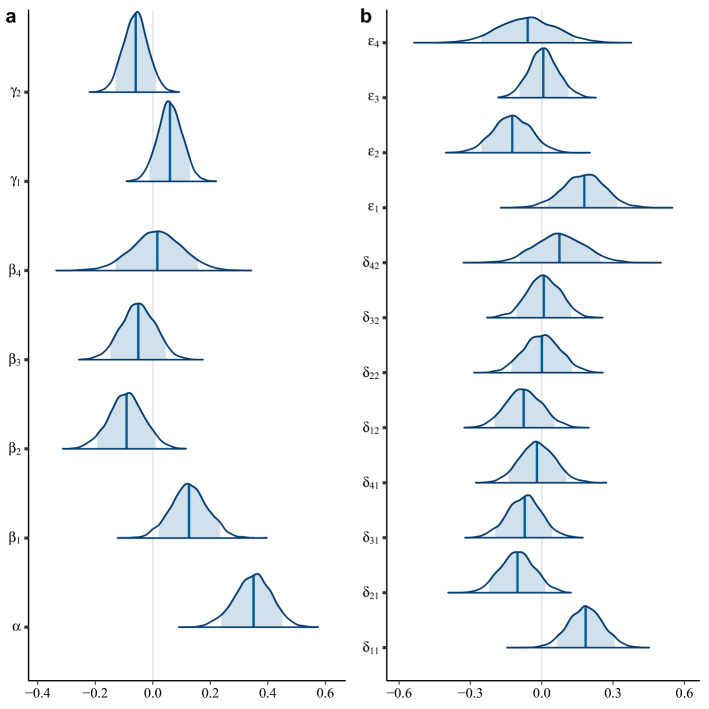
Inferred posterior distributions of regression parameters for all corresponding-group level predictors included in the meta-regression model. The mean and 90% credible interval (5th and 95th quantiles) are highlighted within the posteriors in blue. In panel **a**: α corresponds to the effect of inbreeding level of populations on ID, β is the life stage effect (1-embryonic, 2-juvenile vegetative, 3-adult vegetative, 4-adult reproductive), γ is the effect of species connectivity (1-well-connected, 2-fragmented). In panel **b**: δ is the effect of the interaction life stage × species connectivity, ε is the effect of the interaction inbreeding coefficient × life stage.

Based on the meta-regression analyses using model 2, we first focused on the distribution of ID across inbreeding levels and life stages (and their interactions) of the populations under study. The inbreeding coefficient (denoted F), obtained from the crossing design for each ID estimate, had a strong positive effect on ID (α, Fig. 4, Table S2). In other words, the more inbred a study population, the higher the expected average ID. The 95% CI of the regression coefficient, α, did not contain zero, suggesting that it was beneficial to incorporate crossing design in the analysis through F.

The life stage predictor was observed to have a profound effect on ID (β, Fig. 4, Table S2). ID effects were most positive in the embryonic stage and most negative in the juvenile vegetative growth stage; neither of these uncertainty intervals contained zero (Table S2). Interestingly, of all life stages, only the embryonic stage coefficient was clearly positive, which implies a very strong effect of this life stage on ID. This finding is not surprising as the ID estimates for the earliest stages reported in our raw data were very high, often greater than 0.8 (see e.g. Fig. S1). The adult vegetative and the adult reproductive stages had weak average effects on ID compared to the embryonic and juvenile growth stages, and the 95% CI included zero. However, ID increases with life stage between the juvenile vegetative and final reproductive stages. This trend of increasing levels of ID at later life stages is seen in studies on e.g. *Picea glauca*^24^ and *Pinus taeda*^25^ (Fig. 2).

Furthermore, an interaction term between F and life stage strongly affected ID (*ε*, Fig. 4, Table S2). F had a greater impact on ID at the early embryonic life stage at the 95% significance level, but a reduced impact at the juvenile vegetative growth stage at the 90% significance level. Thus, an increment in F of 0.25 would have very different impact depending on the life stage at which the ID was measured. ID would increase by approximately 0.39 if the study was conducted at the embryonic stage instead of at the juvenile vegetative stage if all other factors were held constant (i.e. the same species and from the same region). For example, Orr-Ewing^26^ reported an increase of 0.35 in ID for *Pseudotsuga menziesii* from inbreeding levels F = 0.5 and 0.75 at the embryonic stage. Thus, the effect of inbreeding level was unevenly distributed across the two first life stages in the *Pinaceae* family.

### Species with continuous areas of distribution are particularly prone to ID at the embryonic life stage

We were particularly interested in whether interactions occurred between life stage and species connectivity, which might indicate that evolutionary forces have acted to alter the mating-system in the *Pinaceae* populations. The effect of the species connectivity (γ, Fig. 4, Table S2) shows that a higher ID was observed in the group with high connectivity, γ_1_, although at a lower level of confidence (90% CI did not contain zero: Fig. 4, Table S2). Species-specific population sizes and genetic differentiation thus influenced the strength of ID.

For interactions between life stage and species connectivity (δ, Fig. 4, Table S2), two of eight interaction group-levels were deemed to be significantly different from zero (i.e. did not include zero at 95% and 90% levels, respectively). The interaction between the early life stage and well-connected groups (*δ*_*11*_) was higher than the estimates for all other δ-group levels, indicating that the main predictors alone could not efficiently capture the profound effect of early ID. Clearly, species connectivity accounted for different early life stage ID (*δ*_*11*_ vs *δ*_*12*_) since the effect of the continuous group was particularly high. The reason for this profound effect was the large estimated ID of > 0.8 reported in several studies across a variety of species, e.g. *Pinus taeda*^27^ and *Picea pungens*^28^ (Fig. 2). In general, if all factors other than species membership in well-connected or fragmented groups were the same in two arbitrary studies, the expected difference in ID would be as large as 0.40, as was often observed (see differences in ID reported in Figs. 1 and 2). To highlight the influence of population connectivity level on ID at the embryonic life stage, we performed predictions of ID for comparison (Fig. S5). Furthermore, the combination of juvenile vegetative life stage and continuous species connectivity (parameter *δ*_21_) resulted in a particularly low ID, indicating that these populations could have been affected by purging at the embryonic stage. This interaction between juvenile vegetative life stage and high species connectivity on ID (δ_21_, Fig. 4) is contributed by a relatively large group of studies in, e.g. *Pinus sylvestris*^29^ and *Pinus taeda*^25^, with reported ID close to zero with n = 10 ID estimates from 9 different studies. Note, however, the outlier ID estimate of 0.71 reported for *Picea abies*^30^.

To summarize the meta-regression analyses, the most important finding indicates that *Pinaceae* populations are particularly vulnerable to ID at the embryonic stage and particularly for species with continuous geographic distributions. Even though all CI contained zero in the fragmented species group (*δ*_12_–*δ*_42_), it is interesting to note that the means of the distributions of *δ* (and also ID) increase from the embryonic to the adult reproductive stages. This trend is more like that expected for inbreeding species.

## Discussion

Species belonging to the *Pinaceae* have some of the highest estimates of lethal equivalents and mutational loads ever reported in the literature^4^, which make these long-lived tree species particularly interesting for longitudinal ID studies. However, because high-quality data from inbreeding experiments are difficult to obtain in long-lived species, meta-analysis of combinations of studies from the literature increases both sample size and power to draw inferences about ID effects at multiple levels. We accounted for several sources of variation that contribute to the level of ID, namely life stage, inbreeding level, species connectivity and importantly, the interactions between species connectivity and life stages and between inbreeding coefficients and life stages. We have revealed the distribution of ID across embryonic, juvenile, adult growth, and reproductive life stages in 18 species of the *Pinaceae* (147 estimates from 41 studies), and shown the effects of species-specific population size and connectivity on ID. To arrive at the final regression model, we evaluated three alternative models to account for species size and connectivity by incorporating 44 published estimates of Fst. A particular novelty of our results is the association between the timing of ID and the connectivity of species; inclusion of the interaction terms in the regression model reduced the between-study heterogeneity considerably. We observed a higher level of ID at the embryonic stage in continuous populations (δ_11_, Fig. 4) than compared to ID at the embryonic stage in fragmented populations (δ_21_, Fig. 4).

It is necessary to recognise several underlying assumptions when interpreting our results. We assumed that the out-crossed, non-inbred reference population used in each study consisted of unrelated trees prior to the inbreeding trials. To reduce biological contributions to bias, we excluded studies having open-pollinated reference populations so that the inbreeding coefficients in the study populations should be as predicted by theory^31^. However, the population histories of the sampled trees in each individual study may have resulted in some level of relatedness, and consequently, our assumption of a total absence of consanguinity may be incorrect. For example, historically small population sizes cause genes to drift, reducing genetic variation. This reduction in genetic variance increases the likelihood of mating between related individuals and inbreeding in a population. As a result, the actual difference in inbreeding coefficients between the control and inbred populations might be lower than what is assumed here, which, in turn, might cause an underestimation of the effect of the inbreeding coefficient on ID. This would make it difficult to detect a further reduction in population fitness following experimental selfed crosses. This phenomenon, known as the baseline hypothesis^13^ induces uncertainty when interpreting reduced ID in small, fragmented populations as a result of purging. In addition, competition for light, nutrients, water and pollen might intensify the effect of inbreeding on the fitness of individual trees over time, a conclusion drawn in several meta-analytic studies^32–34^ and in pedigree analyses of natural animal populations^35–37^. However, Yun and Agrawal^38^ showed that inbreeding depression was more strongly correlated with density dependence (i.e. competition) than with stressful conditions in *Drosophila melanogaster*, while Sandner and Matthies^39^ showed that most stressful treatments decreased ID (i.e. nutrient limitations) in *Silene vulgaris*. See Willi et al.^40^ for a review of the subject.

In species that have been outcrossing for much of their evolutionary history, the progeny of self-pollinations are expected to suffer severe early ID due to the accumulation of recessive lethals^4^. This is the case in the family *Pinaceae*, where early ID, often measured as proportion of seed abortion, is typically high due to high genetic load of recessive alleles exposed as homozygotes upon selfing^41–43^. Husband and Schemske^5^ reviewed the effects of stage-specific ID in both self- and cross-fertilizing species and found that outcrossing species suffered greater ID at early, seed viability stages than selfing species, a pattern that we also observed in our study. Our results also agree with previous findings in conifers and support much higher ID at the earliest developmental stages^17,18,24^ (*β*_1_, Fig. 4). Our data do not, however, support a clear increase in ID towards reproductive life stages (*β*_2_–*β*_4_), indicating that if mutations of mild effect are maintained in later life stages, their effects on ID were not strong enough to be detected.

We utilized Fst information from the literature to partition our species into two groups in which populations were either well-connected over larger areas or occurred as smaller isolated fragments. Species with well-connected distributions had lower estimated Fst values (Fst = 0.241 (0.028)) than those with fragmented distributions (Fst = 0.037 (0.001)), providing a plausible way to model the effect of species-specific population distributions on ID. The levels of ID found in well-connected and fragmented species groups (i.e. *γ,* a main effect in the regression model) showed marginally significant differences. An interesting difference emerged when the interactions between connectivity and life stage were assessed (*δ*, Fig. 4). Populations of species with high connectivity suffered more severely from inbreeding effects during the embryonic stage than fragmented populations (*δ*_11_ vs. *δ*_12_). The differences in ID between embryonic and juvenile stages in the well-connected species (*δ*_11_ vs. *δ*_21_) is likely the result of removal of unfit individuals (i.e. seed abortion), whereas the significantly lower ID observed at the embryonic stage in the fragmented group (*δ*_12_ vs. *δ*_12,_ Fig. 4) may be due to purging that has occurred on site in previous generations. If purging has occurred, decreased ID during the embryonic stage could drive a transition from an outbreeding mating system, typical of well-connected populations, towards selfing in species with fragmented distributions.

The evolutionary transition from predominantly outcrossing to selfing is known in conifers^44^. Large, outcrossing populations maintain substantial ID that acts as a major factor opposing a transition to selfing^18,45^. When an outcrossing population becomes inbred to a sufficient magnitude over time, selection will favour a selfing mating system as long as the strength of ID is maintained below a threshold level of 0.5^46^. A number of theoretical and empirical studies have also stressed that the transition toward selfing in long-lived outcrossed organisms with typically high levels of gene flow is only likely to be initiated where demographic events, such as small population sizes associated with isolation or bottlenecks, act as catalysts^18,45,47,48^. This is because the transition to selfing requires that selfing individuals survive and reproduce over many generations, which is unlikely to occur in well-connected populations.

Using a theoretical approach, Hedrick et al*.*^49^ investigated the effects of multiple genetic factors on the number of lethal equivalents observed in the northern and southern Scots pine populations in Finland^50^. The authors concluded that the reduction in the magnitude of ID in the northern populations may be the result of increased levels of self-fertilization. Furthermore, Vogl et al*.*^20^ found evidence for the evolution of the mating system in native populations of *Pinus radiata*, known to have a history of population bottlenecks, which restricted the species to five small populations. They concluded that purging of early ID was the primary reason for the high proportion of selfed adult trees. Similar findings have been reported in small fragmented populations of *Pinus strobus*^21^, *Pinus resinosa*^22^, *Picea omorika*^23^ , *Pinus contorta*^51^, and *Pinus albicaulis*^52^. To summarize the findings in the literature, large differences in mating system and distribution of ID have been detected both within and between populations as functions of population characteristics such as effective size, isolation by distance and demographic history. Hence, it seems likely that the association between the distribution of species and ID found in our analyses could follow from the aforementioned characteristics, but on a wider geographic scale.

We conclude that even if the lower fitness of inbred populations relative to their outcrossing counterparts might be explained by their presumed smaller effective population sizes (high ID baseline), we cannot reject the potential action of purging, which could drive the evolution of the mating system away from predominant outcrossing in the *Pinaceae.* That the mating system could transition towards increased selfing may be an evolutionary advantage in low density conifer forests or in marginal populations (see Restoux et al*.*^53^). Reproductive assurance accompanying the ability to self might allow colonization of a wider range of environments, a greater tolerance to fluctuations in population size and also allow persistence of *Pinaceae* species populations. Future studies of this topic would benefit from incorporating detailed data on the distributions and demographic histories of the species. Estimates of mating system parameters, such as outcrossing rates of the populations, might also be included in the analyses, as in e.g. Duminil et al*.*^54^.

## Materials and methods

### Sampling

We searched for literature using the Web of Science database for studies of *Pinaceae* species published in peer-reviewed journals. We have also included a number of studies from the forest genetics literature representing older, but valuable, work published in conference proceedings and institutional reports of breeding institutes. All studies included in the final data set were required to fulfil the criteria described in the following sections. In short, a study needed to report: (1) an estimate of ID directly or indirectly, via family mean performance of inbred and non-inbred trees, (2) a measure of deviation around the mean ID estimate either reported directly as standard deviation, variance or coefficient of variance, or indirectly but computable from data in the study.

If ID was reported for multiple populations with the same treatment combination (i.e. same species, life stage and inbreeding level), we chose to include only one estimate (with the lowest standard error) to avoid between-population biases^50^. Because between-family variation in fitness is typically large in inbred populations^18,55^, studies with inbred and control populations that consisted of fewer than three families were not included. When the fitness of each family for inbred and out-crossed populations were only available in graphs, we used Data Theft 3 (http://datathief.org/) to estimate ID. To estimate the standard error in ID when only errors of the phenotypes of different families in the populations were reported, a first order Taylor series approximation was used. Because so few studies have reported within-family variation in fitness, we only acknowledged the between-family variation in our analyses (as standard errors).

### Definition of ID

The distribution of ID across life stages was examined by considering stage specific estimates obtained from each population. Following Husband and Schemske^5^, we define stage specific ID at life stage *y*_*i*_ = 1 − (*w*_*oi*_/*w*_*si*_), *i* = 1,2,3,4, where *w*_*oi*_ and *w*_*si*_ are the fitnesses of the outcrossed and inbred populations, respectively.

The inbred populations represent four classes of crossing designs: half-sib, full-sib, one and two generations of selfing, which result in inbreeding coefficients, F = 0.125, 0.25, 0.5, 0.75, respectively. The advantage of only using populations derived from controlled crosses is that more precise measures of inbreeding are obtained than can be estimated from indirect measures such as outcrossing rate from molecular marker data^56^. Backcross and full-sib matings were treated in the same class of crossing design since both have an average inbreeding coefficient of F = 0.25. Similarly, populations obtained from one generation of selfing and one generation of selfing and additional full-sib crosses, were treated as a crossing design with F = 0.5. We rejected studies with open-pollinated control populations since these contain an unknown proportion of inbred individuals^4,49,57^ and hence have different levels of F relative to studies where control populations were created by controlled outbreeding. Furthermore, we assume that base populations in each study consisted of unrelated and non-inbred trees, prior to the creation of the inbred population. Even though some material might be sampled from breeding populations selected according to specific breeding objectives, most tree breeding programs worldwide are in their infancy and hence consist of unrelated trees^7^.

### Definition of life stages

We followed Greenwood^58^ to define life stages as (1) embryonic (2) post-embryonic juvenile vegetative, (3) adult vegetative, and (4) adult reproductive stages. The first life stage contained seed quality traits: percentage of sound seeds, sound seeds per cone and total number of sound seeds. Due to parthenocarpy (production of fruit without fertilization of ovules), those individual studies that reported the percentage of sound seeds in *Abies*, *Larix*, *Picea* and *Pseudotsuga*, may contribute to an overestimation of the level of early ID.

The second life stage comprised germination-related traits expressed in the first year after sowing. These traits were germination as percentage of sown seeds, hypocotyl height and germination rate in days. The post-germination stages were divided into pre-reproductive and post-reproductive stages. Since most studies reported either height of the trunk, diameter at breast height and/or survival after planting in the field trial, we included these vegetative traits as measures of fitness, since use of commonly reported traits facilitated a better comparison between species and populations. Stem volume and sectional area were not considered in our analyses because as functions of power of height and diameter, respectively, ID appears to be much higher than those reported for height and diameter^59^. We chose the age of reproduction to be 12 growing seasons so that estimates of ID were placed in either the pre-flowering (embryonic, post-embryonic juvenile vegetative and adult vegetative) or post-flowering (adult reproductive) stages, depending on the age of the population. We acknowledge that it is not strictly accurate to choose a fixed age of reproduction for all species and populations because reproductive age may vary with environmental factors (i.e. climate, water and nutrition supply and competition effects) and the demographic history of the population.

### Modelling the effect of species distribution

We present three alternative ways to model the available species distribution data. In the first model, denoted model 1, estimates of inbreeding effects obtained from species with typically fragmented population distributions were treated as one group while species with large continuous distributions were treated as a second group, based on the species distribution area data from United States Department of Agriculture Natural Resources Conservation Service (http://plants.usda.gov/) for North American species and Agro Forestry Tree Database (http://www.worldagroforestry.org/) for European species. As an alternative to this partitioning, published estimates of F_st_ for each species were collected. We interpret high values of Fst as a sign of high genetic differentiation between subpopulations possibly due to a lack of migration while the opposite is assumed for low values of F_st_ (low levels of genetic differentiation and a substantial gene flow between sub-populations). If a difference between the groups is detected in the meta-regression analysis, we can infer that distribution area, our proxy for population size, explains a portion of the variability in ID estimates. In model 2, as an alternative way to partition the species into two groups, we made use of k-means clustering, based on pair-wise F_st_ between species, implemented in the R software^60^. This method (denoted as model 2) to partition the species minimizes the within-group variability while maximizing the between-group variability in F_st_ and does not require any prior knowledge about the area of distribution. This allows the number of species in each partition to be unbalanced, unlike in model 1. A beta regression model was used to infer the effects of each species on the F_st_ estimate (i.e. the response variable), which is a proportion of the between subpopulation genetic variation and the genetic variation in the total population. This model was implemented following the parametrization suggested by Ferrari and Cribari-Neto^61^. Such a model had the advantage of allowing us to correct for the typical behaviour of a response of proportions and can incorporate the different marker types used to estimate the F_st_. Only F_st_ estimates based on SNPs, nucleus SSR and Isozymes/Allozymes were included. After the regression analysis, we randomly drew 10,000 species effect size values from the inferred posterior of each species and performed k-means clustering for 10,000 repetitions (and thereby acknowledge uncertainty inherited from the regression into the clustering). Interested readers are invited to see https://github.com/jonhar97/Pinaceae-meta-regression for further details into the beta regression analysis including source code. Finally, in model 3, the mean of the obtained posterior of the species effect on F_st_ estimates from the beta regression model were used directly as a covariate in the regression model.

We made use of predictive model selection criteria to identify the preferred regression model (i.e. how to infer the effect of species distribution on ID): leave-one-out cross-validation (LOO) and the LOO information criterion (LOOIC). LOO and LOOIC scores for the included models were computed within the R package ‘loo’^62^.

### Meta-regression model

Our goal is to acknowledge the sources of variation in ID estimates among the set of empirical studies and to infer the effect of life stages and species-specific population distributions on ID. Prior to carrying out the meta-regression analysis, we tested the data for homogeneity following Costa-Font et al.^63^. Under the null hypothesis of homogeneity, Q is distributed as *χ*^2^*N* − 1 where *N* is the sample size (i.e. number of ID estimates). To analyse the meta-regression data set, we modelled inbreeding level as a covariate, since the inbreeding coefficient values are directly comparable between crossing designs. Life stage, species-specific population distribution and an interaction term between life stage and species-specific population distribution were given group-level labels and were thus modelled as categorical factors. This set of terms (predictors) represented the independent variables in the regression model and allow us to account for heterogeneity arising from study specific details, such as study design. The levels of inbreeding were: F = (0.125, 0.25, 0.5, 0.75), and the corresponding regression coefficient is denoted by *α*. Regression coefficients for life stages are denoted by *β*_*l*_, l = 1,…,4 (in the same order as defined in definition of life stages above). These two predictors were identical in all models considered. Species distributions are denoted *γ*_*m*_, m = 1, 2, for well-connected and fragmented distributions, respectively; the interaction term life stage x species connectivity level is denoted *δ*_*lm*_, l = 1,…,4 and m = 1, 2 (for models 1 and 2), and the interaction term inbreeding level x life stage is denoted *ε*_*l*_*,* l = 1,…,4. In model 3, a vector of Fst values was used where each entry corresponds to each ID estimate and the interaction was a combination of the Fst covariate and the life stage factor. The group level effects, a mixture of within- and across-study relationships with inbreeding estimates, are assumed to follow a normal distribution with constant mean and precision. The study effects are denoted by *a*_*j*[*i*]_, j = 1, …,41, and considered to be normally distributed with variance *σ*_*a*_^2^ and with mean centred around zero because an overall intercept is already included in the model, and i is the i:th ID estimate within group j. The species effects are denoted by *b*_*o*[*i*]_, o = 1,…,18, again with mean centred around zero and variance *σ*_*b*_^2^. Thus, both *a*_*j*[*i*]_ and *b*_*o*[*i*]_ are assigned varying intercepts for each group (i.e. study and species, respectively) which allows for correlation in ID within groups. All standard deviation parameters are assigned a uniform prior. As suggested by Smith et al.^64^, the student-t distribution was used as a population distribution for ID in the meta-analysis. Prior for degrees of freedom in Student's t distribution was assumed to follow a gamma (2,0.1) distribution as proposed by Juárez and Steel^65^. The non-nested multilevel meta-regression model we used in the analysis (i.e. models 1 and 2) can be written as:

$$\begin{aligned} & y_{ijklm} \sim t\left( {\Theta_{ijklm} , \, \eta_{ijklm} , \, df} \right), \\ & \Theta_{ijklm} = \mu \, + \, \alpha \, + \, \beta_{l} + \, \gamma_{m} + \, \delta_{lm} + \, \varepsilon_{l} + a_{j[i]} + \, b_{o[i]} , \eta_{ijklm} = \sigma_{y} SE_{ijklm} , \\ & a_{j} \sim N\left( {0,\sigma_{a}^{2} } \right),\;j = 1, \ldots ,41, \\ & b_{o} \sim N\left( {0,\sigma_{b}^{2} } \right),\;o = 1, \ldots ,18, \\ & df\sim gamma\left( {2,0.1} \right), \\ \end{aligned}$$

where *μ* is the overall mean, *SE*_*ijklm*_ is the standard error of the i:th ID estimate, *df* and *η*_*ijklm*_ are the degrees of freedom and weighted standard deviation of the t distribution, respectively. For identifiability of the group-level regression coefficients, a hard constraint was imposed by setting the sum to zero for each set of coefficients belonging to the same predictor: ∑*β*_*i*_ = 0, ∑*γ*_*i*_ = 0, ∑*δ*_*i*_ = 0, and ∑ε_*i*_ = 0, summarized over all group levels *i*. For model 3, *γ*_*m*_ and *δ*_*lm*_ were instead a covariate and a covariate × factor interaction, respectively, with corresponding posterior mean of the Fst predictor for each species as obtained in the beta regression analysis described in the “Modelling the effect of species distribution” section. Two levels of significance of the regression coefficients were defined: significant if the zero was outside the 95% credible interval (CI), and marginally significant if the zero was outside the 90% CI. Predictions of ID were made using the Student-t distribution with the inferred regression coefficients and assuming an average study and species effects as parameters. The regression models were implemented and analysed through the RStan software^66^. Default parameters controlling the collection of Monte Carlo chain samples were used. Forest plots were generated in R^60^ using function forest.plot.or available at: http://www.medicine.mcgill.ca/epidemiology/joseph/pbelisle/forest-plot.html, and modified to add group-level covariates data. Density plots of inferred posterior distributions of all regression coefficients in the model were created using the R-package bayesplot^67^. More information about analysis details and data is provided at https://github.com/jonhar97/Pinaceae-meta-regression, and in Table S1.

## Supplementary information

Supplementary information

## Data Availability

The data are provided in Table S1 and at https://github.com/jonhar97/Pinaceae-meta-regression.
