# Supplementary material for: Life stage-specific inbreeding depression in long-lived Pinaceae species depends on population connectivity
Source: Sci Rep. 2021 Apr 23;11:8834. doi: 10.1038/s41598-021-88128-4 (PMC8065042; doi:10.1038/s41598-021-88128-4)
Supplement: Supplementary file 1 — Supplementary information [file 41598_2021_88128_MOESM1_ESM.pdf]

Supplementary information

Title: Life stage-specific inbreeding depression in long-lived *Pinaceae* species depends on population connectivity

Jon Ahlinder, Barbara E Giles, M Rosario García-Gil

Table S1 Data used in synthesis where number, reference, species, species distribution, inbreeding coefficient, life-cycle stage, trait, estimated stage specific inbreeding depression (y), standard error (SE) of each study is given. For species distribution column, F and C denote fragmented and continuous distribution, respectively. In column life stage, E denotes the embryonic stage, JV denotes the juvenile vegetative stage, AV denotes the adult vegetative stage and AR denotes adult reproductive stage. Traits amount of sound seeds and germination were given as a percentage of the total seeds and plants sown, respectively.

No	Reference	Species	Spec. distr.	Inbr. coeff.	Life-Cycle stage	Trait	y	SE
1	Barnes 1964	*Pinus monticola*	F	0.5	JV	Epicotyl length	0.214	0.152
					AV	Survival	0.230	0.140
						Height	0.310	0.030
2	Barnes et al. 1962	*Pinus monticola*	F	0.5	E	Sound seed yield per cone	0.247	0.190
					AV	Survival	0.308	0.278
3	Bingham 1973	*Pinus monticola*	F	0.75	AV	Survival	0.380	0.091
					E	Sound seed yield	0.774	0.093

						per cone		
4	Bingham and Squillace 1955	*Pinus monticola*	F	0.5	E	Sound seed yield per cone	0.473	0.110
					JV	Germination	0.122	0.213
					AV	Height	0.209	0.074
5	Bishir and Namkoong 1987	*Pinus taeda*	C	0.5	E	Amount of sound seeds	0.818	0.082
		Pinus virginiana	C		E		0.830	0.074
6	Coles and Fowler 1976	*Picea glauca*	C	0.5	E	Amount of sound seeds	0.830	0.074
					AV	Epicotyl length	0.258	0.116
7	Cram 1984	*Picea pungens*	C	0.5	E	Sound seed yield per cone	0.600	0.135
					JV	Germination	0.284	0.106
8	Dengler 1939	*Pinus sylvestris*	C	0.5	AV	Height	0.098	0.092
9	Dieckert 1964	*Picea abies*	C	0.5	E	Amount of sound seeds	0.181	0.368
					JV	Germination	0.707	0.143
		Larix deciduas	F		AV	Survival	0.213	0.206
					AV	Height	0.114	0.129
10	Durel and Kremer 1995	*Pinus pinaster*	F	0.75	AV	Height	0.264	0.026
						Diameter	0.376	0.047
11	Durel et al. 1996	*Pinus pinaster*	F	0.125	AV	Height	0.028	0.027
						Diameter	0.048	0.044
						Survival	-0.001	0.038
				0.25		Height	0.054	0.028
						Diameter	0.100	0.043
						Survival	-0.022	0.034
				0.5		Height	0.153	0.029
						Diameter	0.250	0.042

						Survival	0.072	0.045
				0.75		Height	0.265	0.033
						Diameter	0.370	0.050
						Survival	0.198	0.066
12	Ehrenberg et al 1955	*Pinus sylvestris*	C	0.5	E	Amount of sound seeds	0,526	0,104
					AV	Height	0,194	0,159
13	Fowler and Park 1983	*Picea glauca*	C	0.5	E	Amount of sound seeds	0.865	0.044
					JV	Germination	0.084	0.155
						Cotyledon number	0.016	0.041
					AV	Height	0.310	0.065
						Survival	-0.014	0.036
					AR	Height	0.444	0.057
						Diameter	0.640	0.072
						Survival	0.077	0.044
14	Franklin 1969	*Pinus taeda*	C	0.5	E	Amount of sound seeds	0.716	0.027
					JV	Germination	0.139	0.003
						Hypocotyl height after 6 months	0.074	0.003
						Height after 6 months	0.128	0.200
						Survival after 6 weeks	0.015	0.008
					AV	Survival	0.168	0.028
15	Geburek 1986	*Picea omorica*	F	0.5	AR	Diameter at breast height	0.300	0.030
16	Griffin and Lindgren 1985	*Pinus radiate*	F	0.125	E	Amount of sound seeds	-0.024	0.050
				0.25			0.030	0.035
				0.5			0.566	0.100
				0.75			0.580	0.100
17	Johnsen et al. 2003	*Picea mariana*	C	0.5	AR	Survival	0.610	0.077
						Height	0.378	0.056

						Diameter	0.390	0.054
18	Jonsson 1976	*Pinus sylvestris*	C	0.5	E	Amount of sound seeds	0.749	0.060
19	Kormutak and Lindgren 1996	*Abies alba*	F	0.5	E		0.417	0.138
20	Kormutak et al 2005	*Pinus sylvestris*	F	0.5	E	Amount of sound seeds	0.762	0.053
					JV	Germination	0.012	0.335
21	Kraus and Squillace 1964	*Pinus eliotti*	C	0.5	AV	Height	0.317	0.111
					JV	Germination speed	0.197	0.254
					E	Sound seed yield per cone	0.779	0.140
22	Langner 1959	*Picea omorica*	F	0.5	E	Amount of sound seeds	-0.081	0.210
					JV	Germination	-0.046	0.146
					AV	Height	0.143	0.079
23	Layton and Goddard 1983	*Pinus eliotti*	C	0.125	AV	Height	0.165	0.084
				0.25			0.119	0.074
				0.125		Survival	0.027	0.063
				0.25			0.054	0.049
24	Lindgren 1974	*Picea abies*	C	0.5	AV	Height	0.106	0.115
25	Lindgren 1975	*Picea abies*	C	0.5	AV	Height	0.326	0.142
26	Lundkvist et al. 1987	*Pinus sylvestris*	C	0.5	AR	Height	0.200	0.050
					AV		0.300	0.100
27	Matheson et al. 1995	*Pinus eliotti*	C	0.25	AR	Diameter	0.202	0.074
				0.125			0.123	0.066
				0.25		Height	0.121	0.064
				0.125			0.064	0.077
28	Orr-Ewing 1965	*Pseudotsuga menziesii*	C	0.5	AR	Height	0.296	0.040

						Survival	0.236	0.063
				0.75	E	Amount of sound seeds	0.892	0.053
				0.5			0.539	0.124
				0.75	AV	Height	0.127	0.091
				0.5			0.045	0.079
29	Park and Fowler 1984	*Picea mariana*	C	0.5	AV	Height	0.189	0.047
					E	Amount of sound seeds	0.545	0.118
					JV	Germination	0.074	0.054
					AV	Survival	0.238	0.050
30	Pawsley 1964	*Pinus radiate*	F	0.5	JV	Germination	0.143	0.268
31	Plym-Forshell 1974	*Pinus sylvestris*	C	0.5	E	Amount of sound seeds	0.823	0.052
32	Skröppa and Tho 1990	*Picea abies*	C	0.5	E	Amount of sound seeds	0.717	0.061
33	Skröppa 1996	*Picea abies*	C	0.5	AV	Height	0.330	0.022
34	Snyder and Squillace 1966	*Pinus eliotti*	C	0.5	E	Sound seed yield per cone	0.609	0.175
			C	0.5	E		0.526	0.104
					AV		0.194	0.159
35	Sorensen 1999	*Pseudotsuga menziesii*	C	0.5	AR	Height	0.251	0.028
						Diameter	0.439	0.033
						Survival	0.171	0.057
		Pinus ponderosa	F			Height	0.333	0.036
						Diameter	0.490	0.040
						Survival	0.045	0.072
		Abies procera	F			Height	0.395	0.108
						Diameter	0.474	0.159
						Survival	0.040	0.077
36	Sorensen 2001	*Pinus contorta*	C	0.5	JV	Emergence	-0.054	0.005
					AV	Height	0.244	0.005
						Diameter	0.219	0.005
37	Sorensen 1997	*Pseudotsuga*	C	0.125	AV	Height	0.054	0.039

		menziesii						
						Diameter	0.069	0.036
				0.25		Height	0.151	0.024
						Diameter	0.202	0.028
					JV	Hypocotyl height	0.036	0.010
						Emergence	-0.005	0.010
38	Wang et al 2004	*Pseudotsuga menziesii*	C	0.125	AV	Height	0.117	0.092
				0.25			0.221	0.086
				0.5			0.442	0.079
39	Woods et al 2002	*Pseudotsuga menziesii*	C	0.125	AV	Diameter	0.048	0.033
				0.25			0.046	0.050
				0.5			0.053	0.056
				0.125		Height	0.062	0.036
				0.25			0.055	0.055
				0.5			0.048	0.068
				0.125		Survival	0.183	0.076
				0.25			0.195	0.094
				0.5			0.305	0.131
40	Woods and Heaman 1989	*Pseudotsuga menziesii*	C	0.125	E	Amount of sound seeds	0.314	0.073
				0.25			0.486	0.088
				0.5			0.958	0.015
41	Wu et al. 1998	*Pinus radiate*	F	0.125	AR	Diameter	0.050	0.154
						Survival	-0.030	0.459
				0.25		Diameter	0.060	0.208
						Survival	0.010	0.489
				0.5		Diameter	0.150	0.171
						Survival	0.070	0.448
				0.75		Diameter	0.190	0.233
						Survival	0.110	0.552

Table S2. Summary statistics of the inferred parameters in the meta-regression model using two partitions to account for species geographic distribution. The summary statistics of the inferred study and species effects, $a_{j[i]}$ and $b_{o[i]}$ respectively, are not shown (but see Supplementary Figs. S1 and S2). The regression parameters are: α, the effect of inbreeding coefficient on y, β, the effect of life stage, γ, the effect of species fragmentation level, δ, the interaction between life stage and species fragmentation level, ε, the interaction between inbreeding level and life stage, σ_y is the residual standard deviation, σ_a and σ_b is the standard deviation of the study effect and species effect, respectively.

Parameter	Predictor	Mean	Median	SD	2.5%	97.5%
α	Inbreeding level	0.348	0.349	0.065	0.215	0.473
β_1	Life stage	0.125	0.125	0.066	-0.003	0.255
β_2		-0.088	-0.088	0.061	-0.209	0.036
β_3		-0.052	-0.052	0.058	-0.166	0.061
β_4		0.015	0.014	0.087	-0.155	0.188
γ_1	Species fragmentation	0.060	0.060	0.043	-0.023	0.144
γ_2		-0.060	-0.060	0.043	-0.144	0.023
δ_{11}	Interaction:	0.184	0.184	0.074	0.040	0.330
δ_{21}	Life cycle	-0.104	-0.104	0.072	-0.245	0.038
δ_{31}	Species fragmentation	-0.070	-0.070	0.070	-0.207	0.068
δ_{41}		-0.017	-0.017	0.074	-0.164	0.127
δ_{12}		-0.075	-0.074	0.075	-0.222	0.072
δ_{22}		-0.001	-0.001	0.076	-0.155	0.149
δ_{32}		0.007	0.007	0.069	-0.128	0.1412

δ_{42}		0.078	0.078	0.107	-0.133	0.285
ε_1	Interaction:	0.179	0.179	0.087	0.006	0.348
ε_2	Inbreeding level	-0.124	-0.124	0.081	-0.288	0.033
ε_3	Life stage	0.009	0.009	0.062	-0.109	0.135
ε_4		-0.063	-0.062	0.121	-0.305	0.171
σ_y	Residual stdev	1.275	1.267	0.155	1.001	1.612
σ_a	Study level stdev	0.049	0.048	0.018	0.016	0.088
σ_b	Species stdev	0.030	0.026	0.021	0.002	0.081

Figure S1. ID estimates of species with continuous and fragmented distributions for the group levels: **A** inbreeding coefficients, and **B** life stages.

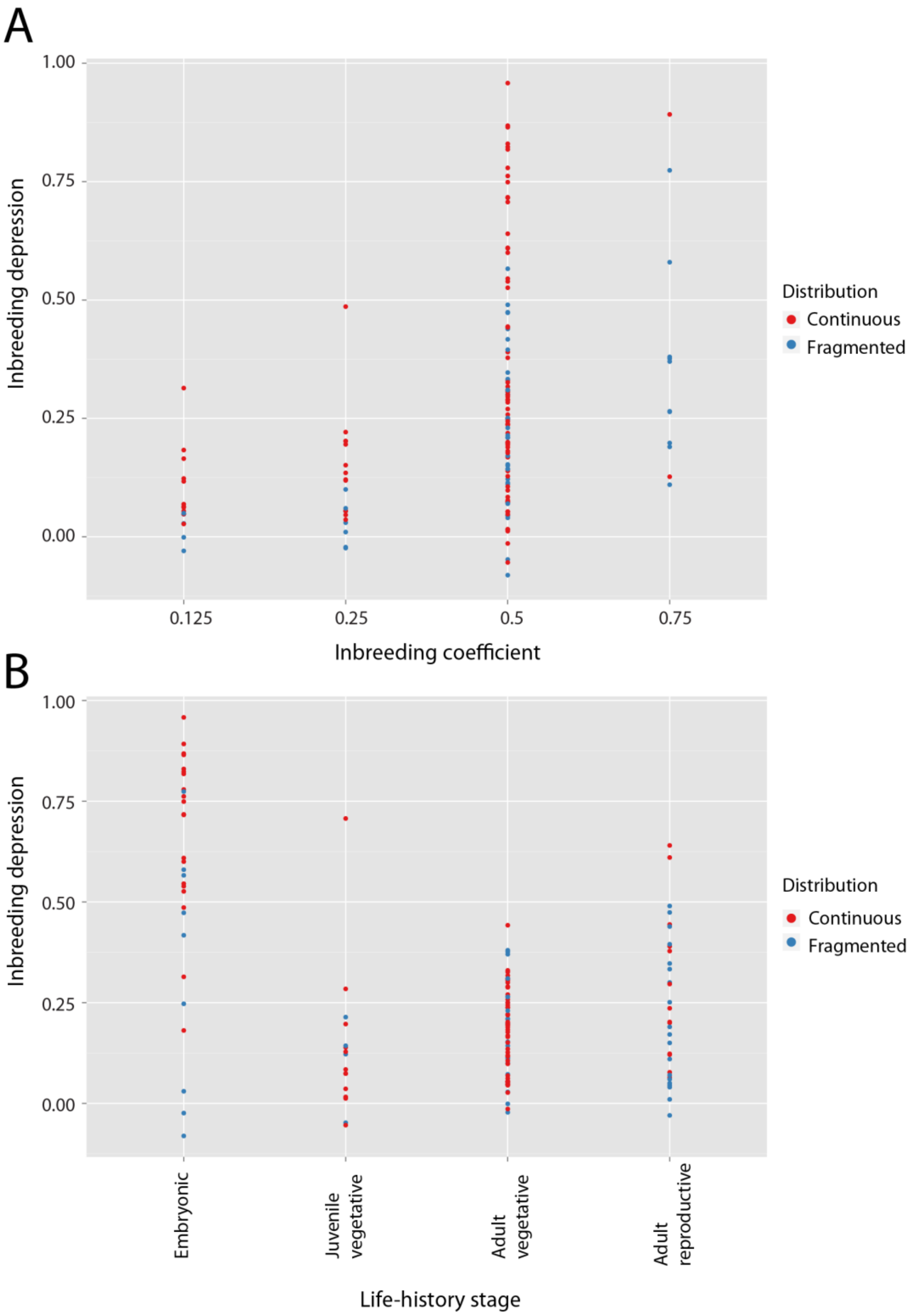

Figure S2. The results of the beta-regression analysis with inferred posterior distributions for the effect of *Pinaceae* species on Fst value with mean and 90% credible interval highlighted with blue colours.

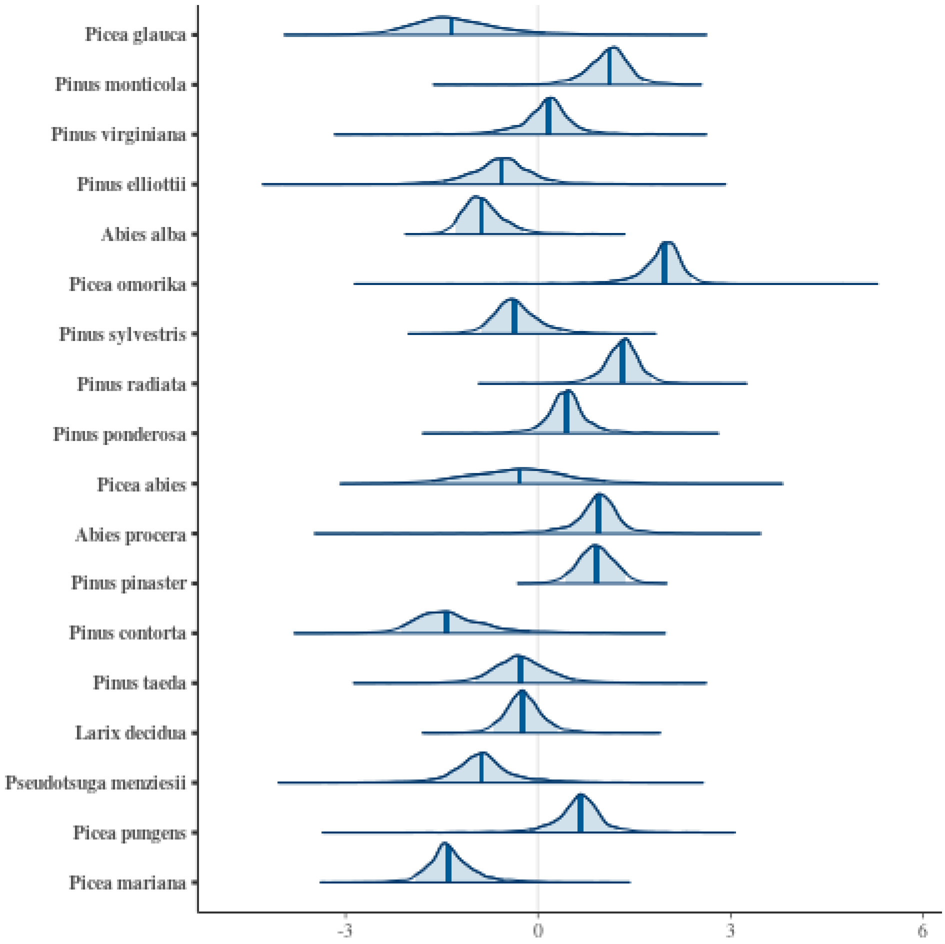

Figure S3. Obtained posterior summary statistics of the species group level factor with mean and 90% credible interval highlighted with blue colours.

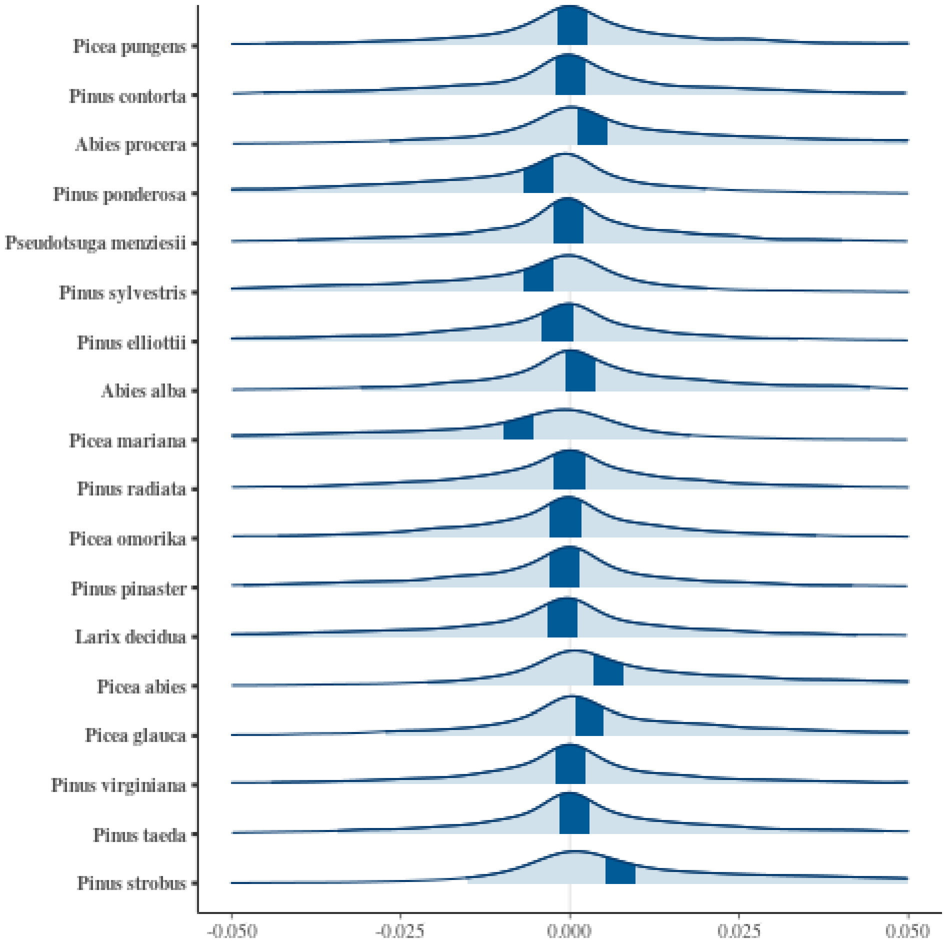

Figure S4. Obtained posterior summary statistics of the study effect factor with mean and 90% credible interval highlighted with blue colours. See Table S1 for the corresponding references of studies 1 to 41, with subfigure **a** for studies 1-20 and **b** for 21-41.

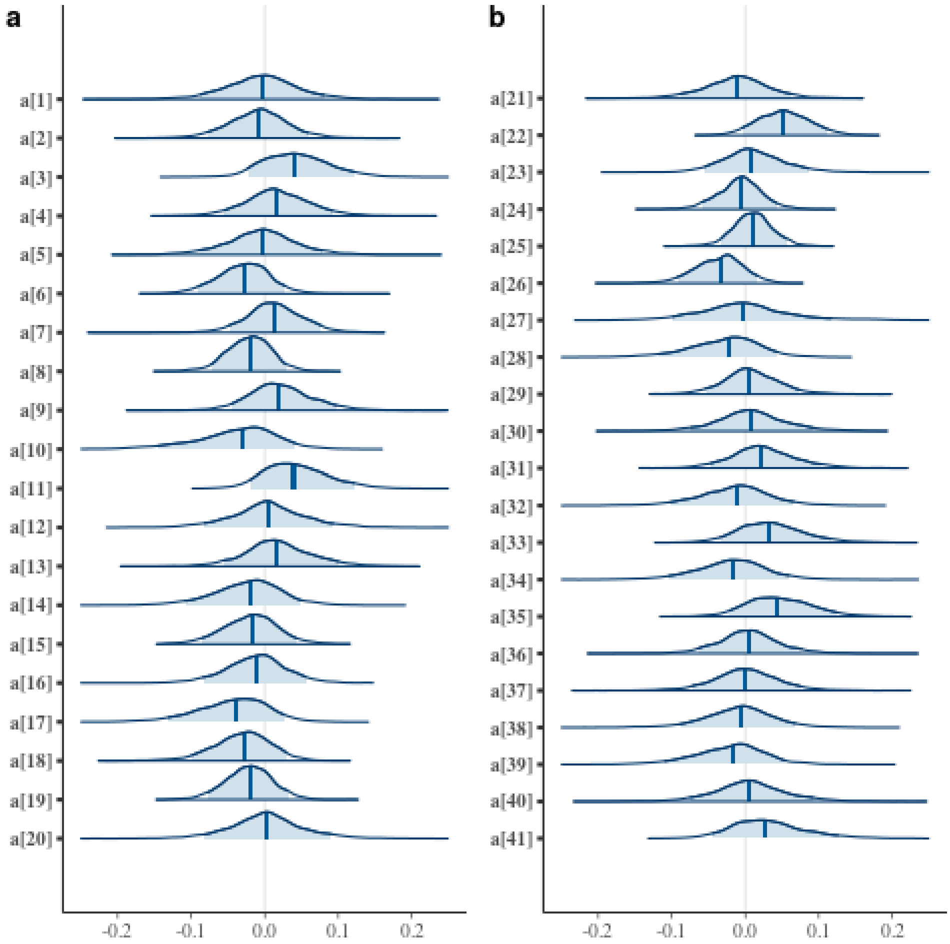

Figure S5. To highlight the difference in early ID in the continuous and fragmented species groups, we performed predictions of the ID across all life stages using the same meta regression model, where the predicted ID is denoted $y_{pred[i,j]}$ for life stage i and species group j) across all life stages for: **a** species with continuous area of distribution, and **b** with fragmented area of distribution. 90% credible interval of the distribution is highlighted in blue. The mean predicted ID at the embryonic stage was 0.74 (0.26) and 0.38 (0.40) for the continuous and the fragmented groups, respectively, with $p(y_{pred[1,1]} > y_{pred[1,2]}) = 0.91$. However, at the later stages, levels of predicted ID were very similar between the groups: 0.31 (0.29) and 0.28 (0.37) at the adult reproductive stage, for example.

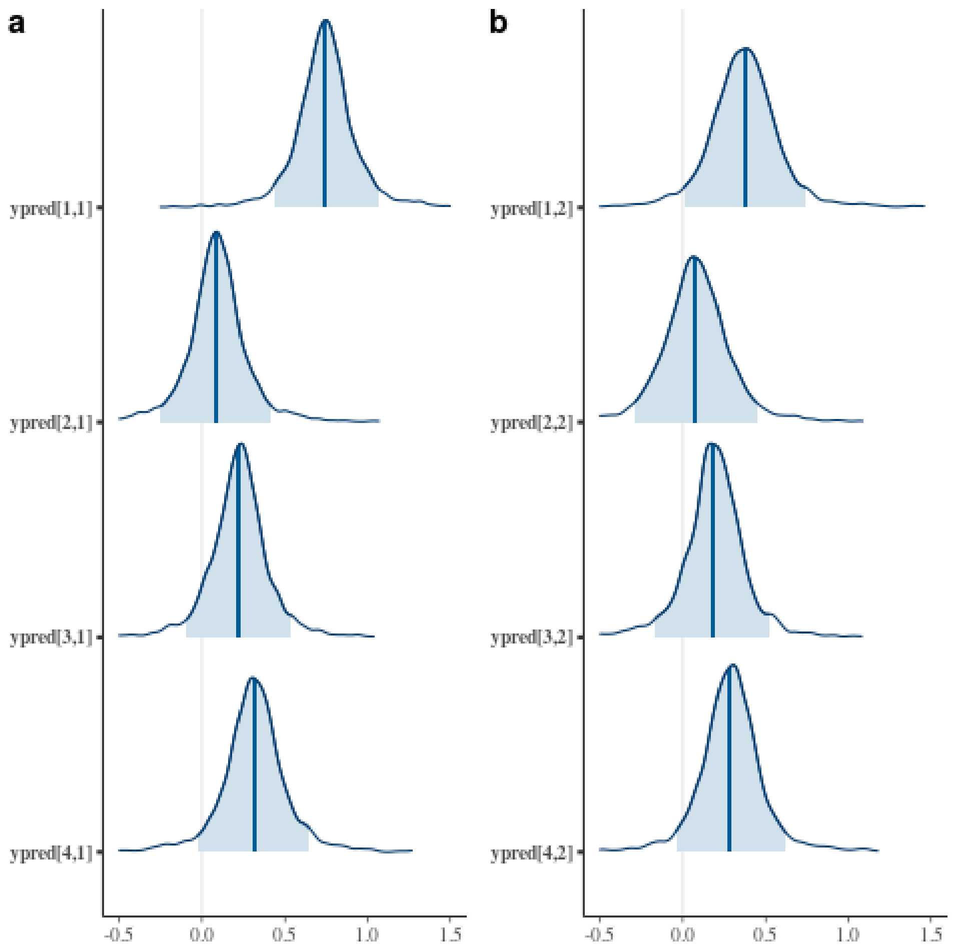

References used to collect the ID data

Barnes BV. **1964**. Self- and cross-pollination of western White pine: a comparison of height growth of progeny. *USDA Forest Service Research Note* **INT-22**.

Barnes BV, Bingham RT, Squillace AE. **1962**. Selective fertilization in *Pinus monticola*. II. Results of additional tests. *Silvae Genetica* **11**: 103-111.

Bingham R. **1955**. Self-compatibility and effects of self-fertility in western white pine. *Forest Science*: 121-129.

Bingham RT. **1973**. Possibilities of improvement of western white pine by inbreeding. *USDA Forest Service Research Papers* **Int-44**.

Bishir J, Namkoong G. **1987**. Unsound seeds in conifers - estimation of numbers of lethal alleles and of magnitudes of effects associated with the maternal parent. *Silvae Genetica* **36**: 180-184.

Coles JF, Fowler D P. **1976**. Inbreeding in neighboring trees in two White Spruce populations. *Silvae Genetica* **25**: 29-34.

Cram WH. **1984**. Some effect of self-pollination, cross-pollination, and open-pollination in *Picea pungens*. *Canadian Journal of Botany* **62**: 392-395.

Dengler A. **1939**. Über die Entwicklungen künstlicher Kiefernkreuzungen. *Zeitschrift fur Forst- und Jagdwesen* **71**: 457-485.

Dieckert H. **1964**. Some investigations on self sterility and inbreeding in Spruce and Larch. *Silvae Genetica* **13**: 77-86.

Durel C E, Kremer A. **1995**. Hybridization after self-fertilization: a novel perspective for the maritime pine breeding program. *Forest Genetics* **2**: 117-120.

Durel C. E., Bertin P, Kremer a. 1996. Relationship between inbreeding depression and inbreeding coefficient in Maritime pine (*Pinus pinaster*). *Theoretical and Applied Genetics* **92-92**: 347-356.

Ehrenberg CE, Gustafsson A, Plym-Forshell C, Simak M. **1955**. Seed quality and the principles of forest genetics. *Hereditas* **41**: 291-366.

Fowler D. P., Park Y. S. 1983. Population studies of White spruce. I. Effects of self-pollination. *Canadian Journal of Forest Research* **13**: 1133-1138.

Franklin EC. **1969**. Inbreeding as a means of genetic improvement of Loblolly pine. Proceedings of the Tenth Southern Conference on Forest Tree Improvement. Huston, Texas, 107-115.

Geburek T. **1986**. Some results of inbreeding depression in Serbian spruce (*Picea omorika* (Panc) Purk.). *Silvae Genetica* **35**: 169-172.

Griffin AR, Lindgren D. **1985**. Effect of inbreeding on production of filled seed in *Pinus radiata* - experimental results and a model of gene action. *Theoretical and Applied Genetics* **71**: 334-343.

Johnsen K, Major JE, Maier C a. **2003**. Selfing results in inbreeding depression of growth but not of gas exchange of surviving adult black spruce trees. *Tree physiology* **23**: 1005-8.

Jonsson H. **1976**. Contributions to the genetics of empty grains in the seed of Pine (*Pinus silvestris*). *Silvae Genetica* **25**: 10-15.

Kormutak A, Lindgren D. **1996**. Mating system and empty seeds in silver fir (*Abies alba* Mill.). *Forest Genetics* **3**: 231-235.

Kormutak A, Ostrolucka M, Vookova B, Pretova A, Feckova M. **2005**. Artificial hybridization of *Pinus sylvestris* L. and *Pinus mugo* Turra. *Acta Biologica Cracoviensia Series Botany* **47**: 129-134.

Kraus JF, Squillace AE. **1966**. Selfing vs. outcrossing under artificial conditions in P*inas elliottii* Engelm. *Silvae Genetica* **13**: 72-76.

Langner W. **1959**. Selbstfertilität und Inzucht bei *Picea omorica* (Pancic) Purkyne. *Silvae Genetica* **8**: 84-93.

Layton PA, Goddard RE. **1983**. Low level inbreeding effects on germination, survival, and early height growth of slash pine. Proceedings of the Southern Forest Tree Improvment Conference 17.106-115.

Lindgren D. **1974**. Aspects on suitable number of clones in a seed orchard. Population and Ecological Genetics, Breeding Theory and Progeny testing, Proceedings of the IUFRO Joint Meeting. Stockholm, 293-305.

Lindgren D. **1975**. *Rapport angående inavelsdepression i fytotronen med gran*. Stockholm.

Lundkvist K, Eriksson G, Norell L, Ekberg I. **1987**. Inbreeding depression in two field trials of young *Pinus sylvestris* L. *Scandinavian Journal of Forest Research* **2**: 281-290.

Matheson AC, White TL, Powell GR. **1995**. Effects of inbreeding on growth, stem form and rust resistance in *Pinus elliottii*. *Silvae Genetica* **44**: 37-46.

Orr-Ewing A. **1965**. Inbreeding and single crossing in Douglas-fir. *Forest Science* **11**: 279-290.

Park Y S, Fowler D P. **1982**. Effects of inbreeding and genetic variances in a natural population of tamarack (*Larx laricina* (Du Roi) K. Koch) in Eastern Canada. *Silvae Genetica* **31**: 21-26.

Park Y S, Fowler D P. **1984**. Inbreeding in Black Spruce (*Picea-Mariana* (Mill) Bsp) - Self-Fertility, Genetic Load, and Performance. *Canadian Journal Of Forest Research* **14**: 17-21.

Pawsey CK. **1964**. *Inbreeding radiata pine (Pinus Radiata D. Don.)*. Canberra: Forest and Timber Bureau.

Plym-Forshell C. **1974**. Seed development after self-pollination and cross-pollination of Scots pine, *Pinus sylvrstris* L. *Studia Forestalia Suecia* **118**: 1-24.

Skröppa T. **1996**. Diallel crosses in *Picea abies*. II. Perdormance and inbreeding depression of selfed families. *Forest Research*.

Snyder EB, Squillace AE. **1966**. Cone and seed yield from controlled breeding of southern pines. *South Forest Experimental Station, U. S. Forest Service Research Papers* **22**.

Sorensen F. **2001**. Effect of population outcrossing rate on inbreeding depression in *Pinus contorta* var. *murrayana* seedlings. *Scandinavian Journal of Forest Research* **16**: 391-403.

Sorensen FC. **1997**. Effects of sib mating and wind pollination on nursery seedling size, growth components, and phenology of Douglas-fir seed-orchard progenies. *Canadian Journal of Forest Research* **27**: 557-566.

Wang T, Aitken SN, Woods JH, Polsson K, Magnussen S. **2004**. Effects of inbreeding on coastal Douglas fir growth and yield in operational plantations: a model-based approach. *Theoretical and applied genetics* **108**: 1162-1171.

Woods J, Wang T, Aitken S. **2002**. Effects of Inbreeding on Coastal Douglas-fir : Nursery Performance. *Silvae Genetica* **314**: 430-436.

Woods JH, Heaman J. **1989**. Effect of different inbreeding levels on filled seed production in Douglas-fir. *Canadian Journal of Forest Research* **19**: 54-59.

Wu HX, Matheson AC, Spencer D. **1998**. Inbreeding in *Pinus radiata*. I. The effect of inbreeding on growth, survival and variance. *Theoretical and Applied Genetics* **97**: 1256-1268.

References from which the *Fst* estimates in the *Pinaceae* were obtained (see Table 1 in the main text).

Achere V, Favre JM, Besnard G, Jeandroz S. 2005. Genomic organization of molecular differentiation in Norway spruce (*Picea abies*). *Mol Ecol* **14**: 3191–3201.

Ballian D, Longauer R, Mikić T, Paule L, Kajba D, Gömöry D. 2006. Genetic structure of a rare European conifer, Serbian spruce (*Picea omorika* (Panč.) Purk.). *Plant Syst Evol* **260**: 53-63.

Berg EE, Hamrick JL. 1997. Quantification of genetic diversity at allozyme loci. *Can J For Res* **27**: 415-424.

Chen J, Källman T, Ma X, Gyllenstrand N, Zaina G et al. 2012. Disentangling the roles of history and local selection in shaping clinal variation of allele frequencies and gene expression in Norway spruce (*Picea abies*). *Genetics* **191**: 865-881.

Csillery K, Ovaskainen O, Sperisen C, Widmer A, Gugerli F. 2019. Adaptation to local climate in a multi-trait space: evidence from silver fir (Abies alba Mill.) populations across a heterogeneous environment. *bioRxiv* 292540.

Cvjetković B., Konnert M, Fussi B, Mataruga M, Šijačić-Nikolić M, et al. 2017. Norway spruce (*Picea abies* Karst.) variability in progeny tests in Bosnia and Herzegovina. *Genetika* **49**, 259- 272.

Dvornyk V, Sirviö A, Mikkonen M, Savolainen O. 2002. Low nucleotide diversity at the pal1 locus in the widely distributed *Pinus sylvestris*. *Mol Biol Evol* **19**: 179–188.

Eckert AJ, Bower AD, González-Martínez SC, Wegrzyn JL, Coop G, et al. 2010. Back to nature: ecological genomics of loblolly pine (*Pinus taeda*, Pinaceae). *Mol Ecol* **19**: 3789-3805.

Eveno E, Collada C, Guevara MA, Léger V, Soto A, Díaz L, Garnier-Géré PH. 2008. Contrasting Patterns of Selection at Pinus pinaster Ait. Drought Stress Candidate Genes as Revealed by Genetic Differentiation Analyses. *Mol Biol Evol* **25**: 417–437.

Giovannelli G, Scotti-Saintagne C, Scotti I, Roig A, Spanu I, et al. 2019. The genetic structure of the European black pine (Pinus nigra Arnold) is shaped by its recent Holocene demographic history. *bioRxiv* 535591

González-Díaz P, Jump AS, Perry A, Wachowiak W, Lapshina E, Cavers S. 2017. Ecology and management history drive spatial genetic structure in Scots pine. *Forest Ecology and Management* **400**: 68-76.

Jaramill-Correa J-P, Rodríguez-Quilón I, Grivet D, Lepoittevin C, Sebastiana F, Heuertz, M, Garnier-Géré P, Alia R, Plomion C, Vendramin GG, Gonzalez-Martinez, S. C. 2015. Molecular proxies for climate maladaptation in a long-lived tree (*Pinus pinaster* Aiton, Pinaceae). *Genetics* **199**: 793–807.

Karhu A, Hurme P, Karjalainen M, Karvonen P, Kärkkäinen K, Neale D, Savolainen O. 1996. Do molecular markers reflect patterns of differentiation in adaptive traits of conifers? *Theor Appl Genet* 93: 215-221.

Karhu A, Vogl C, Moran GF, Bell JC, Savolainen O. 2006. Analysis of microsatellite variation in *Pinus radiata* reveals effects of genetic drift but no recent bottlenecks. *J Evol Biol* **19**: 167-175.

Kinloch B, Westfall RD, Forrest GI. 1986. Caledonian Scots pine: origins and genetic structure. *New Phytologist* 104: 703-729.

Latta RG, Mitton JB. 1999. Historical separation and present gene flow through a zone of secondary contact in ponderosa pine. *Evolution 53*: 769-776.

Ledig FT, Capo-Arteaga MA, Hodgskiss PD, Sbay H, Flores-Lopez C, et al. 2001. Genetic diversity and the mating system of a rare Mexican pinon, Pinus pinceana, and a comparison with Pinus maximartinezii (Pinaceae). *Am J Botany* **88**: 1977-1987.

Ledig FT, Hodgskiss PD, Johnson DR. 2006. The structure of genetic diversity in Engelmann spruce and a comparison with blue spruce. *Can J Bot* **84**: 1806-1828.

Lesser MR, Parchman TL, Buerkle CA. 2012. Cross-species transferability of SSR loci developed from transciptome sequencing in lodgepole pine. *Mol Ecol Res* **12**: 448-455.

Liu J-J, Sniezko RA, Ekramoddoullah AKM. 2011. Association of a novel *Pinus monticola* chitinase gene (PmCh4B) with quantitative resistance to Cronartium ribicola. *Phytopathology* **101**: 904-911.

Kim M-S, Richardson BA, McDonald GI, Klopfenstein NB. 2011. Genetic diversity and structure of western white pine (*Pinus monticola*) in North America: A baseline study for conservation, restoration, and addressing impacts of climate change. *Tree Genet Genom* **7**: 11–21.

Matusova R. 1995. Genetic variation in 5 populations of silver fir (*Abies alba* Mill.) in Slovakia. *Biologia (Bratislava)* **50**: 53–59.

Moran CF, Bell JC, Eldridge KG. 1988. The genetic structure and the conservation of the five natural populations of *Pinus radiata*. *Can J For Res* **18**: 506-514.

Mosca E, Eckert AJ, Di Pierro EA, Rocchini D, La Porta N, Belletti P, Neale DB. 2012. The geographical and environmental determinants of genetic diversity for four alpine conifers of the European Alps. *Molec Ecol* **21**: 5530–5545.

Namroud M-C, Beaulieu J, Juge N, Laroche J, Bousquet J. 2008. Scanning the genome for gene single nucleotide polymorphisms involved in adaptive population differentiation in white spruce. *Mol Ecol* **17**: 3599–3613.

Parchman TL, Gompert Z, Mudge J, Schilkey FD, Benkman CW, et al. 2012. Genome-wide association genetics of an adaptive trait in lodgepole pine. *Mol Ecol* **21**: 2991-3005.

Parker K, Hamrick J, Parker A, Stacy E. 1997. Allozyme diversity in *Pinus virginiana* (Pinaceae): intraspecific and interspecific comparisons. *Am J Bot* **84**: 1372-1382.

Plomion C, Bartholomé J, Lesur I, Boury C, Rodríguez- Quilón I, et al. 2016. High- density SNP assay development for genetic analysis in maritime pine (Pinus pinaster). Mol Ecol Resour, 16: 574-587.

Pyhäjärvi T, García-Gil MR, Knürr T, Mikkonen M, Wachowiak W, Savolainen O. 2007. Demographic history has influenced nucleotide diversity in European Pinus sylvestris populations. *Genetics* **177**: 1713–1724.

Roschanski AM, Csilléry K, Liepelt S, Oddou- Muratorio S, Ziegenhagen B, et al. 2016. Evidence of divergent selection for drought and cold tolerance at landscape and local scales in Abies alba Mill. in the French Mediterranean Alps. *Mol Ecol*, **25**: 776-794.

Saenz-Romero C, Guries RP, Monk AI. 2001. Landscape genetic structure of *Pinus banksiana*: allozyme variation. *Can J Bot* **79**: 871-878.

Šarac Z, Aleksić J, Dodoš T, Rajčević N, Bojović S, Marin P. 2015. Cross-species amplification of nuclear EST-microsatellites developed for other Pinus species in Pinus nigra. *Genetika*, **47**: 205-217.

Scalfi M, Piotti A, Rossi M, Piovani P. 2009. Genetic variability of Italian southern Scots pine (*Pinus sylvestris* L.) populations: the rear edge of the range. *Eur J For Res* **128**: 377-386.

Soto A, Robledo-Arnuncio JJ, González-Martínez SC, Smouse PE, Alía R. 2010. Climatic niche and neutral genetic diversity of the six Iberian pine species: a retrospective and prospective view. *Mol Ecol* **19**: 1396-1409.

Viard F, El-Kassaby Y-A, Ritland K. 2001. Diversity and genetic structure in populations of *Pseudotsuga menziesii* (Pinaceae) at chloroplast microsatellite loci. *Genome* **44**: 336–344.

Unger GM, Konrad H, Geburek T. 2012. Does spatial genetic structure increase with altitude? An answer from *Picea abies* in Tyrol, Austria. *Plant Syst Evol* **292**: 133-141.

Wachowiak W, Iason GR, Cavers S. 2013. Among population differentiation at nuclear genes in native Scots pine (Pinus sylvestris L.) in Scotland. *Flora* **208**: 79-86.

Wagner S, Gerber S, Petit RJ. 2012. Two highly informative dinucleotide SSR multiplexes for the conifer *Larix decidua* (European larch). *Mol Ecol Resour* **12**: 717-25.

Wang ZM, Macdonald SE. 1992. Peatland and upland black spruce populations in Alberta, Canada: Isozyme variation and seed germination ecology. *Silvae Genet* **41**: 117-122.

Ye TZ, Yang R-C, Yeh FC. 2002. Population structure of a lodgepole pine (*Pinus contorta*) and jack pine (*P. banksiana*) complex as revealed by random amplified polymorphic DNA. *Genome* **45**: 530-540.

Yeh FC, Hu XS. 2005. Genetic structure and migration from mainland to island populations in *Abies procera* Rehd. *Genome* **48**: 461-473.
